# Detrimental Effect of Water Submersion of Stools on Development of *Strongyloides stercoralis*

**DOI:** 10.1371/journal.pone.0082339

**Published:** 2013-12-16

**Authors:** Witthaya Anamnart, Attarat Pattanawongsa, Pewpan Maleewong Intapan, Nimit Morakote, Penchom Janwan, Wanchai Maleewong

**Affiliations:** 1 Department of Medical Technology, School of Allied Health Sciences and Public Health, Walailak University, Nakhon Si Thammarat, Thailand; 2 Department of Preclinical Science, School of Pharmacy, Walailak University, Nakhon Si Thammarat, Thailand; 3 Department of Parasitology and Research and Diagnostic Center for Emerging Infectious Diseases, Faculty of Medicine, Khon Kaen University, Khon Kaen, Thailand; 4 Department of Parasitology, Faculty of Medicine, Chaing Mai University, Chaing Mai, Thailand; University of Houston, United States of America

## Abstract

Strongyloidiasis is prevalent in Thailand, yet its prevalence in the south is lower than in other parts of the country. This might be due to the long rainy season in the south resulting in stool submersion in water inhibiting worm development. In this study, the effect of water submersion of fecal samples on development of *Strongyloides stercoralis* was investigated. Ten ml of a 1∶5 fecal suspension were placed in 15-ml tubes, 35-mm dishes, and 90-mm dishes producing the depths of 80 mm, 11 mm and 2 mm-suspensions, respectively. The worm development was followed at 1/6, 4, 6, 8, 10, 12, 14, 16, 24, and 36 h, by determining the number of filariform larva (FL) generated from agar-plate cultures (APC). Fecal suspensions kept in tubes and 35-mm dishes showed a decline in FL yield relative to incubation time and reached zero production 14 h after incubation. In contrast, the number of FL generated from the suspension kept in 90-mm dishes remained stable up to 36 h. Cumulatively, all tubes and 35-mm dishes became negative in APC after 14 h while 90-mm dishes remained APC-positive up to 36 h. Adding more water or stool suspension to dishes resulted in a decreased number of FL. Mechanical aeration of the suspensions in tubes restored an almost normal FL yield. It appears that the atmospheric air plays a significant role in growth and development of *S. stercoralis* in the environment and may be one of factors which contribute to a lower prevalence of human strongyloidiasis in the south of Thailand.

## Introduction

Strongyloidiasis, a harmful infectious disease for immunosuppressed patients caused by *Strongyloides stercoralis*, is estimated to occur in 30-100 million people worldwide [1]. Prevalence data from 1990 to 2010 showed that *S. stercoralis* infection rates in community detected by stool culture ranged from 15.9% to 38.8% in northern [2], [3], 23.5% to 28.9% in northeastern [4], [5], [6] and 30.5% in Bangkok, central Thailand [7]. However, a recent estimate in southern Thailand ranged from 1.8% to 10% which was lower than expected [8], [9], [10]. Despite hookworm and *S. stercoralis* infect human via skin penetration, the ratio of strongyloidiasis to hookworm infection in Bangkok was 1.20∶1 [7] and in northeastern was 2.35∶1 [6], in contrast, that in southern only ranged from 0.07∶1to 0.19∶1 [8], [9], [10]. Factors affecting different in distribution may include good hygiene practices among population and availability of sewerage system. Climate factor, interestingly, may be one of the factors as rainy season was longer in the south. These differences raise questions that environmental factors which affect transmission of the worm may be different among regions. It was then postulated that rainfall may play an important role as southern Thailand has a long lasting rainy season for 9 to 10 months whereas other regions have only a 4 months-long rainy season. Areas continuously covered with surface water for some period may be adverse to worm growth and development. In accordance with this postulation, unexpected findings in a previous study revealed that the number of rhabditiform larva (RhL) per gram of stool was decreased when specimens were left in saline for up to 6 hours [11]. Lost larvae were found dead and trapped in the debris plug after subjection to the formalin-ether sedimentation technique. A preliminary experiment also showed that mixing *S. stercoralis*-positive stool with distilled water or saline at 1∶5 and left standing for 6 hours was detrimental to survival of RhL as reflected by negative agar plate cultures.

The laboratory experimental study was therefore designed to demonstrate that prolonged submersion of stools in water was detrimental to growth and development of RhL of *S. Stercoralis*.

## Materials and Methods

### Ethics statements

Patients provided their written informed consent to participate in this study. The ethics committees approved this consent procedure. This study was approved by the Ethics Committee on Human Rights Related to Research Involving Human Subjects, Walailak University.

### Stool samples

Stool samples were collected from chronic strongyloidiasis cases in Thasala District of Nakhon Si Thammarat, southern Thailand. The samples were selected according to the following criteria (a) agar plate culture generated a large number of filariform larva (FL), (b) a simple direct stool smear showed at least 1 motile RhL, and (c) the RhL count by the modified formalin-ether concentration technique [11] revealed 50–200 RhL per gram of stool, (d) other parasites were not detected in stool, and (e) all patients had not been treated with any anthelmintic drugs prior to stool collection.

### Development of *S. stercoralis* upon varying depth of stool suspensions

Stool specimens were processed within 2 hours of defecation. A specimen from each of the 30 total patients was processed as follows. Two grams of stool was placed in a 15-ml sterile graduated conical tube containing 8 ml of distilled water. The content was mixed well by stirring with a wooden stick, producing a 1∶5 stool suspension. More preparations were similarly prepared and transferred to either 35-mm plastic dishes (6-well plates) or 90-mm plastic Petri dishes. Depths of stool suspensions in the 3 containers were 80, 11 and 2 mm, respectively. In the dishes, stool suspensions covered about 2/3 of its bottom area. Ten replicates were performed on each specimen and container size. All tubes and dishes were loosely covered with the lids and left standing at room temperature (26°C–31°C and 70–85% relative humidity). At 1/6, 4, 6, 8, 10, 12, 14, 16, 24, and 36 hours, the stool suspension in each container was subjected to agar-plate culture (APC). For dishes, the contents were transferred to 15-ml conical centrifuge tubes. Dishes were checked for remaining larvae or growing adult worms by stereo microscopy, if found, these were transferred to the corresponding tubes. Tubes were centrifuged at 700×*g* for 5 min. The supernatants were decanted and the fecal sediments were transferred to the center of nutrient agar plates for APC as described below. The tubes were washed with 5 drops of distilled water and transferred to corresponding stool sediment on nutrient agar plates.

### Effect of fecal suspension depths on development of *S. stercoralis*

A 1∶5 stool suspension from each of 6 patients was distributed to three 90-mm dishes. One dish was a control, the second dish had 60 ml of water added to produce a 12 mm-depth suspension, and the third dish had an additional 60 ml of the stool suspension added. Growth and development of worms were followed at intervals as described above up to 18 hours.

### Restoration of growth and development of *S. stercoralis* by aeration

A 1∶5 stool suspension from each of 6 patients was distributed to two 15-ml tubes and two 50-ml tubes. One of two tubes served as a control and was left standing at room temperature. Another tube had a small flexible plastic pipe tip placed in the bottom of the suspension and was continuously aerated. The aeration was accomplished with the use of a fish tank aquarium air pump. Worm growth and development was evaluated at intervals as described above.

### Agar plate culture (APC)

APC was performed as previously described [12]. Briefly, fresh stool samples or stool pellets after centrifugation of the fecal suspensions, were placed at the center of nutrient agar plates and incubated at room temperature for 5 days. Then, 10 ml of 10% formalin was added to the agar surface of each dish and aspirated with a pipet. The collected suspensions were centrifuged and the supernatant fluid was removed so that only 2 ml remained. A drop of the suspension was placed on a microscope slide and the larvae were counted under the microscope. The larval recovery for each plate was then calculated and expressed as number of FL. Species were confirmed under the microscope.

### Statistical Analyses

Descriptive statistics, including means, standard deviations (SD) and ranges were generated by using Microsoft Office Excel 2007. SPSS 14.0 for Windows was used to perform the statistical analyses. A *P* value of <0.05 was considered statistically significant.

## Results

### Development of *S. stercoralis* in different containers

The number of FL generated from the stool suspension kept in tubes at an 80 mm-depth decreased rapidly from 10 min onwards (Table 1). FL numbers in 35-mm dishes at an 11 mm-depth showed a similar pattern except having the higher number of larvae. In addition, 33.3% of samples in tubes failed to generate FL when kept for 6-hour while 93.3% of samples in 35-mm dishes also failed 10 hour after incubation (Table 2). When left for 14-hours or more, both of them completely failed to give FL in APC (Table 2). In contrast, FL output generated from APC of the fecal suspension kept in 90-mm dishes remained high up to 36 hours of incubation and then began to drop about 7.5 fold (Table 1). Nonetheless, none of the fecal suspensions from the 90-mm dishes resulted in negative numbers in APC throughout the 36 hours incubation period (Table 2). Beyond this point, FL numbers decreased at 48 hours and approached zero at 60 hours (data not shown).

**Table 1:** Growth and development of *S. stercoralis* in stool suspensions kept in different containers.^a^

Incubation time (hours)	No. of FL recovered from APC^b^			
	15-mm Tube	35-mm Petri dish	90-mm Petri dish	*P* c
1/6	9,807.3±8,214.3 (650–26,400)	9,887.0±8,251.8 (600–27,000)	9,920.0±8,341.6 (630–26,900)	0.999
4	4,200.3±4,080.7 (120–15,000)	5,693.7±5,336.2 (320–19,000)	9,416.3±7,982.3 (600–26,000)	0.022
6	714.7±1,108.4 (0–4,500)	852.0±1,001.7 (10–4,800)	9,646.0±8,251.8 (600–26,400)	<0.001
8	50.0±132.9 (0–560)	97.1±140.6 (5–550)	9,827.7±8,474.7 (600–27,400)	<0.001
10	0.6±2.4 (0–12)	1.3±5.2 (0–25)	9,844.0±8,276.0 (650–26,400)	<0.001
12	0.17±0.91 (0–5)	0.17±0.91 (0–5)	9,959.0±8,387.2 (700–27,000)	<0.001
14	0 (0)	0 (0)	9,749.0±8,134.1 (700–27,000)	<0.001
16	0 (0)	0 (0)	9,592.3±7,870.7 (700–26,000)	<0.001
24	0 (0)	0 (0)	9,025.7±7,399.2 (700–25,000)	<0.001
36	0 (0)	0 (0)	1,257.3±1,207.9 (100–4,000)	<0.001

±8,287.8 (650–26,900) FL.^a^ Fresh stool produced 10,058

^b^ Means and standard deviation (range) derived from 30 different stool samples.

^c^ Nonparametric Kruskal-Wallis test was analyzed.

**Table 2:** Growth and development of *S. stercoralis* in different containers.^a^

Incubation time (hours)	Cumulative no. of samples negative for FL following APC^b^		
	15-ml Tube	35-mm Petri dish	90-mm Petri dish
1/6	0 (0%)	0 (0%)	0 (0%)
4	0 (0%)	0 (0%)	0 (0%)
6	10 (33.3%)	0 (0%)	0 (0%)
8	24 (80%)	0 (0%)	0 (0%)
10	28 (93.3%)	28 (93.3%)	0 (0%)
12	29 (96.7%)	29 (96.7%)	0 (0%)
14	30 (100%)	30 (100%)	0 (0%)
16	30 (100%)	30 (100%)	0 (0%)
24	30 (100%)	30 (100%)	0 (0%)
36	30 (100%)	30 (100%)	0 (0%)

^a^ Total numbers derived from 30 different stool samples.

%).^b^ No. (

### Effect of stool suspension depths on growth and development of *S. stercoralis*

Table 3 shows that adding excess water or stool suspensions to existing stool suspensions in the 90-mm dishes was detrimental to worm development. Addition of water to produce a final 12-mm depth resulted in decreased FL at 6 h and no FL at 8 h after incubation. Adding stool suspensions to produce the same depth was worse in that no FL were generated after 6 h of incubation.

**Table 3:** Effect of the depth of fecal suspensions on production of FL.^a^

Incubation time (hours)	No. of FL recovered from APC^b^			
	No water adding	Water added to 12- mm depth	Stool suspension added to 12-mm depth	*P* c
1/6	1,471.0±621.0 (695–2,290)	Not done	Not done	NA^d^
6	1,373.3±577.0 (730–2,200)	20.0±49.0 (0–120)	0 (0)	0.001
8	1,328.3±562.4 (700–2,200)	0 (0)	Not done	0.002
12	1,305.0±527.4 (790–2,140)	0 (0)	0 (0)	<0.001
18	1,166.7±478.2 (650–1,850)	0 (0)	Not done	0.002

±674.1 (800–2,500) FL.^a^ Fresh stool produced 1,561.7

^b^ Means and standard deviation (range) derived from 6 different stool samples.

^c^ Nonparametric Kruskal-Wallis test was analyzed.

^d^ Not applicable.

### Restoring worm growth and development by aeration

Table 4 summarizes the results of aeration of the fecal suspensions. Control tubes without mechanical aeration yielded very low numbers of FL at 6 h and no FL at 8 h and further incubation. On the contrary, tubes provided with mechanical aeration yielded a steady number of FL throughout the 18 hours period of incubation.

**Table 4:** Effect of aeration of stool suspensions on production of FL.^a^

Incubation time (hours)	No. of FL recovered from APC^b^					
	15 ml-tube			50 ml-tube		
	No aeration	With aeration	*P* c	No aeration	With aeration	*P* c
1/6	1,484.5±636.0 (715–2,314)	Not done	NA^d^	1,462.5±614.0 (675–2,300)	Not done	NA^d^
6	39.2±61.7 (0–135)	1,280.0±521.0 (680–2,100)	0.002	45.0±70.4 (0–150)	1,381.7±579.5 (700–2,250)	0.002
8	0 (0)	1,266.7±570.7 (700–2,200)	0.003	0 (0)	1,430.0±621.0 (650–2,320)	0.001
12	0 (0)	1,216.7±505.6 (670–2,000)	0.002	0 (0)	1,365.0±566.2 (700–2,200)	0.001
18	0 (0)	1,093.3±449.5 (600–1,750)	0.002	0 (0)	1,326.7±526.9 (750–2,000)	0.001

±674.1 (800–2,500) FL.^a^ Fresh stool produced 1,561.7

^b^ Means and standard deviation (range) derived from 6 different stool samples.

^c^ Unequal variance t-tests were analyzed.

^d^ Not applicable.

## Discussion

*Strongyloides* is a facultative parasite having both homogonic and heterogonic cycles. Outside the human host, RhL in stools may develop directly to infective FL (homogonic cycle) or indirectly to free-living adult males and females which mate and produce RhL progeny and develop finally into FL in the heterogonic cycle [13], [14], [15]. High temperatures of more than 15°C have been demonstrated to favor heterogonic or the indirect free-living cycle of *S. ratti* [16], [17]. Other influential environmental factors are not clear. Defecation on the ground and the tropical climate of southern Thailand may therefore enhance the indirect cycle, increase FL in the soil environment and, as a result, increase the transmission potential of *S. stercoralis*.

Steady yields of FL from stool suspensions kept in 90-mm dishes up to 24 hours suggests that in a certain environment where spreading of stools in a thin layers with enough moisture is optimal for RhL development in the stools to survive and enter the indirect life cycle. On the contrary, excess water or thicker stool sediments are detrimental to RhL development as demonstrated by poor FL output upon storage of stool suspensions in tubes or 35 mm-dishes for 6 hours or more. RhL in the stool suspensions kept in tubes exposed to the most severe, adverse environment caused RhL to fail to survive. This is reflected by absence of FL from APC after short storage in tubes, i.e., in just 14 hours. The finding is supported by the design of Harada-Mori and slant cultures for detection of *S. stercoralis* [18]. In these methods, stool is spread on a filter paper in which its one end dips into the water. The stool stayed above water all the time, yet it got moisture from the water. RhL entered the heterogonic cycle and many FL were generated and observed in the water after 10 days [19].

Decreased RhL survival under experimental conditions is proposed to be due to deprival of atmospheric oxygen or any in atmospheric air. This was confirmed by restoration of FL production by mechanically aerating of stool suspensions in tubes. The requirement of atmospheric air is indirectly supported by the charcoal culture for production of *Strongyloides* FL in which spaces between charcoal granules provided natural aeration. Contradictory to the atmospheric air requirement, development of eggs of *S. fülleborni* in thin stool smears has been demonstrated to require increasing carbon dioxide while decreasing oxygen [20]. It is noteworthy that in the present experience, submerging a piece of stool under water for 6–14 h caused a loss in FL production (data not shown). This finding suggests that RhL and adults are susceptible to atmospheric air deprivation. Once they become FL, they survive even in the water. Further experiments should be done to validate oxygen or any in atmospheric air requirement for the hetergonic development of *S. stercoralis*.

The present results show the detrimental effect of water submersion on development of *S. stercoralis*. However, more analyses are needed to prove our hypothesis.
